# Evaluation of photography using head-mounted display technology (ICAPS) for district Trachoma surveys

**DOI:** 10.1371/journal.pntd.0009928

**Published:** 2021-11-08

**Authors:** Fahd Naufal, Christopher J. Brady, Meraf A. Wolle, Michael Saheb Kashaf, Harran Mkocha, Christopher Bradley, George Kabona, Jeremiah Ngondi, Robert W. Massof, Sheila K. West

**Affiliations:** 1 Dana Center for Preventive Ophthalmology, Wilmer Eye Institute, Baltimore, Maryland, United States of America; 2 Larner College of Medicine, University of Vermont, Burlington, Vermont, United States of America; 3 Kongwa Trachoma Project, Kongwa, Tanzania; 4 Ministry of Health–Community Development, Gender, Elderly and Children, Dodoma, Tanzania; 5 RTI International, Washington DC, United States of America; University of Buea, CAMEROON

## Abstract

**Background:**

As the prevalence of trachoma declines worldwide, it is becoming increasingly expensive and challenging to standardize graders in the field for surveys to document elimination. Photography of the tarsal conjunctiva and remote interpretation may help alleviate these challenges. The purpose of this study was to develop, and field test an Image Capture and Processing System (ICAPS) to acquire hands-free images of the tarsal conjunctiva for upload to a virtual reading center for remote grading.

**Methodology/Principal findings:**

This observational study was conducted during a district-level prevalence survey for trachomatous inflammation—follicular (TF) in Chamwino, Tanzania. The ICAPS was developed using a Samsung Galaxy S8 smartphone, a Samsung Gear VR headset, a foot pedal trigger and customized software allowing for hands-free photography. After a one-day training course, three trachoma graders used the ICAPS to collect images from 1305 children ages 1–9 years, which were expert-graded remotely for comparison with field grades. In our experience, the ICAPS was successful at scanning and assigning barcodes to images, focusing on the everted eyelid with adequate examiner hand visualization, and capturing images with sufficient detail to grade TF. The percentage of children with TF by photos and by field grade was 5%. Agreement between grading of the images compared to the field grades at the child level was kappa = 0.53 (95%CI = 0.40–0.66). There were ungradable images for at least one eye in 199 children (9.1%), with more occurring in children ages 1–3 (18.5%) than older children ages 4–9 (4.2%) (χ^2^ = 145.3, *p*<0.001).

**Conclusions/Significance:**

The prototype ICAPS device was robust, able to image 1305 children in a district level survey and transmit images from rural Tanzania to an online grading platform. More work is needed to improve the percentage of ungradable images and to better understand the causes of disagreement between field and photo grading.

## Author summary

Trachoma is the leading infectious cause of blindness worldwide, caused by the bacterium *Chlamydia trachomatis*. Programs targeting trachoma elimination in endemic regions largely rely on periodic prevalence surveys to monitor progress, but training field graders requires active cases, which is becoming challenging as prevalence declines. Photography of the tarsal conjunctiva with remote interpretation via telemedicine may serve as a more auditable, effective, and cost-efficient method for surveys. We developed and evaluated the Image Capture and Processing System (ICAPS), a smartphone-based, hands-free, head-mounted camera system (Samsung Galaxy S8 with custom app, Samsung Gear VR headset, and a Bluetooth-linked foot pedal trigger). The ICAPS was easy to use in challenging field conditions, was able to upload images from Tanzania and link images to field data. The percentage of TF was 5% by both field grade and photo grade, with agreement kappa = 0.53. Additional field training and enhanced certification of photographers may help reduce the proportion of ungradable images; further research on reasons for mismatch of grades between field and photo is needed.

## Introduction

Trachoma is the leading infectious cause of blindness worldwide, caused by repeated bouts of conjunctivitis from the *Chlamydia trachomatis(Ct)* bacterium [1]. The World Health Organization (WHO) is targeting trachoma for elimination as a public health problem by 2030. In order to validate elimination, WHO requires countries to carry out population-based prevalence surveys at district level documenting sustained reduction of trachomatous inflammation—follicular (TF) below 5% in children ages 1–9 years [2]. These surveys rely on standardized field graders to examine the everted upper eyelid for evidence of TF. However, as the prevalence of trachoma declines worldwide, standardizing graders for surveys to document elimination becomes increasingly more expensive and difficult, often involving training in other countries to ensure exposure to enough cases of TF [3]. A recent survey in Laos required flying graders to Ethiopia to conduct training, for example [4]. Because thousands of impact and surveillance surveys will need to be done by countries in endemic and formerly endemic districts to meet the elimination target, solving the problem of grader standardization is urgent.

One potential solution uses telemedicine to acquire and transmit high-quality images of the everted lid to a central reading center to determine the presence or absence of TF. An ideal system would have the following characteristics: 1) be usable efficiently in the field without increasing staffing needs; 2) not be a conduit for transmission through contamination of the camera system; 3) link the images with other data, such as age and gender, collected in the field; 4) the data on prevalence of trachoma would be comparable to data from field grading. Other considerations might include the cost efficiency compared to current surveying practices and the acceptability to trachoma programs and communities. Imaging systems in use largely in research context have consisted of expensive single lens reflex cameras that require at least two individuals to take a picture of the tarsal conjunctiva, or cell phone cameras that without modification do not appear to take comparable images to the SLR cameras.

We have developed an Image Capture and Processing system (ICAPS) that permits hands-free image acquisition of the tarsal conjunctiva, which is linked via barcode to field data. This device allows photographers to take, store and upload the images to an online grading platform that has the potential to eliminate field grading. The ICAPS is comprised of 1) hardware: a Samsung Gear VR headset, and Samsung Galaxy S8 smartphone, paired via Bluetooth with a 3-D printed foot pedal trigger; and 2) software, a custom-built application (app) for bar code reading, bar code labelling of images and imaging as described below.

The two objectives of this study were to first, create and pilot test the ICAPS, and secondly to field test the ICAPS in children ages 1–9 years who are already enrolled in a district-wide impact survey. During the field test, we planned to determine the following metrics:

- robustness of the system under field conditions;
- the proportion of eyes that had gradable images; and
- the TF agreement between image grades compared with those rendered in the field by standardized survey graders.

## Methods

### Ethics statement

This study was approved by the Johns Hopkins School of Medicine Institutional Review Board. The study in Tanzania was approved by the Tanzania National Institute for Medical Research, as part of the district survey. The University of Vermont (UVM) Institutional Review Board deemed the image analysis components of the research performed by the UVM team were not human subjects research and exempt from IRB review. Informed written consent was obtained from all participants’ parents or legal guardians. This study fully adhered to the tenets of the Declaration of Helsinki.

### The ICAPS

**Fig 1:**
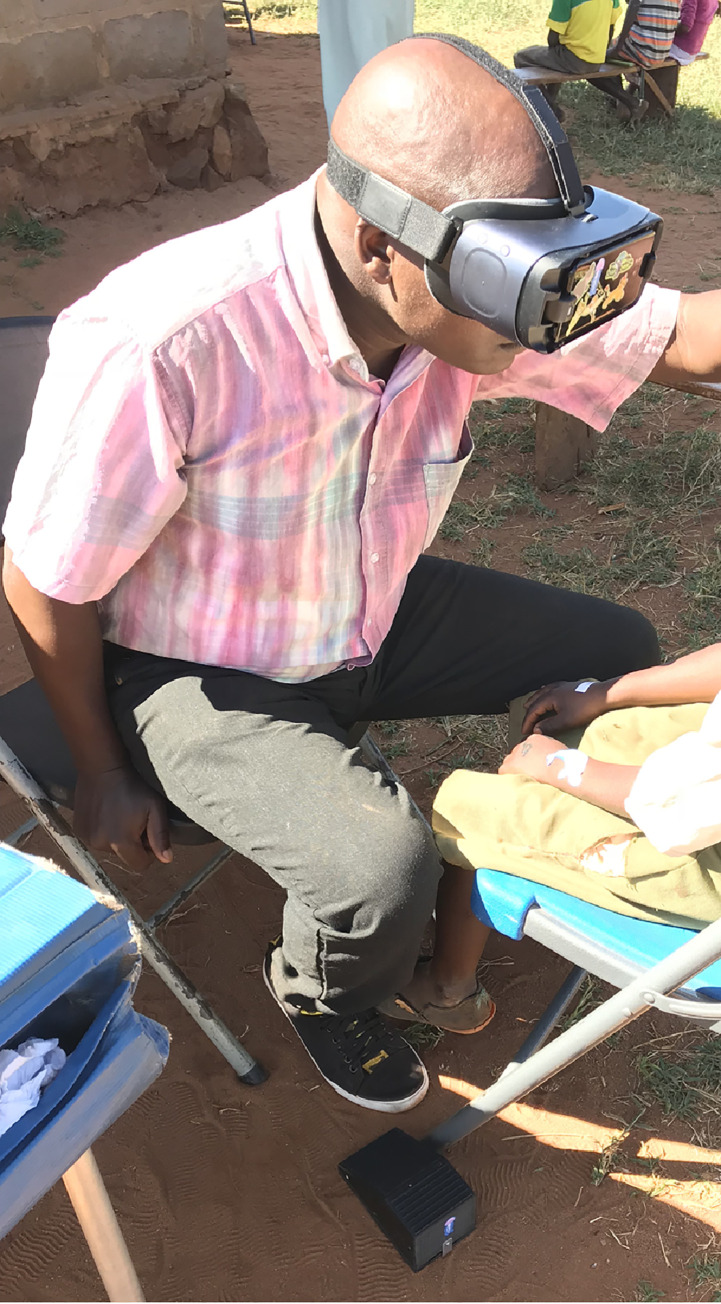
Samsung VR headset and Samsung Galaxy 8 smartphone.

**Fig 2:**
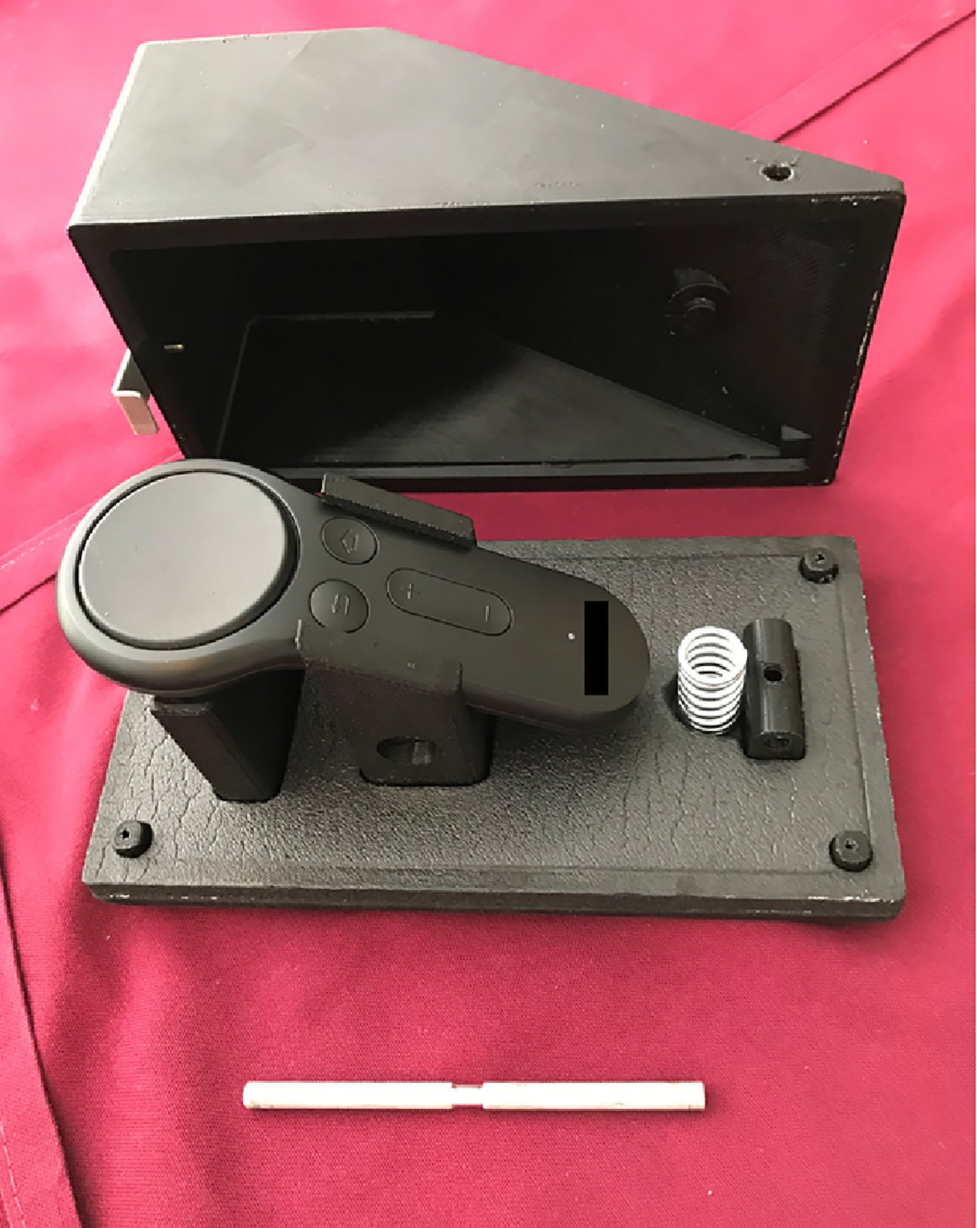
Remote controller inside foot pedal.

The device is a Samsung Galaxy S8 smartphone (rear camera: 12MP, f/1.7, 26mm (wide), 1/2.55” sensor size, 1.4 μm pixel size) inserted into a Samsung Gear VR headset (Samsung Group, Suwon, South Korea) that once worn tracks the movements of the head (Fig 1). The view through the headpiece mimics 2.5X loupes, so the magnified eyelid is seen centrally while still allowing visualization peripherally to maintain proprioception while using the hands to evert the lid. A Bluetooth-paired Gear remote controller was placed inside a 3-D-printed foot pedal and worked as a foot-operated trigger to take photographs (Fig 2). This configuration allowed the photographer to take the image without using hands, thus avoiding contamination of the equipment after everting the lid.

We used Universal Product Code (UPC) standard barcodes as identifiers for each child and the subsequent set of eyelid images. The custom ICAPS application allows the photographer to use subtle head movements to control an on-screen cursor to make selections on the screen to input the bar code, identify the eye as being right or left prior to being photographed, and to preview the image before deciding to save or delete it. The high-resolution images (4032 x3024 pixels) selected for saving are stored in the phone for upload later to a reading center platform.

The headset is powered by the phone and extra pocket-size battery packs were supplied to enable prolonged use in field conditions. The battery packs were able to charge the phones while moving between houses or while in use, if necessary. Of note, the device was put into “sleep mode” when removed from the head to conserve energy.

### Test evaluation of the ICAPS

#### Pilot test at Wilmer eye institute

Images of 41 eyes of 22 volunteers were taken by an ophthalmologist (MW) with experience using ICAPS. All images were graded immediately after the exercise for clarity of the image.

#### Pilot field test in Tanzania

Sixty children ages 1–9 were enrolled in a village known to have prevalent trachoma. A standardized field grader using ICAPS assigned a grade to the eye in the field and took the image. An ophthalmologist (MW) wearing conventional 2.5X loupes also assigned field grades to the eye masked to the grade given by the standardized field grader. Images were downloaded onto a flash drive and the field grader and ophthalmologist graded the images independently. The grading sheets for field and images were entered by a data entry person using Excel for Microsoft Office 365 (Microsoft Corporation, Redmond, WA). The analyses of agreement using kappa statistic was done using SAS 9.4 Software (SAS Institute Inc, Cary, NC).

### Field evaluation of the ICAPS

We performed a field test of the ICAPS system in connection with a district-level impact survey in Chamwino, Tanzania, by integrating with a routine trachoma survey team. This team of standardized graders and data recorders used the Tropical Data mHealth application to record and transmit TF and demographic data separately from the images [5,6].

The survey sample comprised a random sample of 30 clusters (villages) and 25 randomly selected households per cluster. Each grader-recorder pair was assigned to survey a cluster per day and aimed to examine people aged 1 year and above for trachoma signs. Only children aged 1–9 years were eligible for conjunctival image capture using the ICAPS system.

#### District survey training

Three trachoma field graders who had completed a refresher course in grading TF were selected by the Tanzania survey supervisors. The field graders were allotted one day of a training course to become ICAPS photographers. The first half of the day was spent on classroom training to get familiar with using the equipment. The second half was conducted in a village on the same day, where each field grader practiced using the system taking images of children. The field graders needed to take 5 good quality images from 5 different children to become certified photographers for the ICAPS project. After training, the three ICAPS trainers accompanied the three teams to observe implementation in the field.

#### District survey field test in Tanzania

The grader/photographer wearing the ICAPS headgear assessed each eligible child for TF and took photographs of each everted upper eyelid. The recorder scanned the ICAPS UPC into the Tropical Data smartphone to permit data linkage in addition to entering the usual assessment data. One of the trainers uploaded the images from each ICAPS at the end of each day to the online grading platform. Initially, the photographer was allowed to take two images per eye if needed, but this was stopped after the second day and only one image was allowed per eye.

### Image upload and grading for the district field test

We developed a web-based tool to compress and upload the images to a cloud server. We used the Amazon Mechanical Turk crowdsourcing platform to create a customized online grading interface which was used by a single master grader (Senior image grader at the Dana Center with several years of experience grading conjunctival images for trachoma using the WHO simplified grading system, and who was not a part of the field teams) masked to the field grade, village, any individual data, as well as grade of the fellow eye photo. Images were presented randomly and not grouped by participant ID. In the case of 2 images per eye, the images could later be ordered by time stamp for analyses (see below). The goal was to categorize all the images as “TF,” “No TF” or “Ungradable.” A single grader was used for this project and was able to grade at an average rate of 110 images/hour.

### Data management and analyses

The following information collected via Tropical Data was used in our analyses: child age, sex, TF field grade by eye, cluster number, bar code as scanned (or manually entered) by the Tropical Data smartphone. There was no identification for team number or dates for the individual cluster survey. The ICAPS data library consisted of the UPC as scanned by ICAPS, date and time the image was taken. All images were graded and duplicate images for the same eye manually resolved.

The field and photo data were linked, and the proportion of ungradable images was calculated. If an eye had more than one image taken, either the first gradable image was used for analyses, or if none were gradable, the eye was said to have ungradable images. The agreement at eye level between the field grade and the photo grade was calculated using the kappa statistic for the presence or absence of TF. We also determined the agreement between field and photo TF grades at the child level using the kappa statistic. A child was said to have TF in the field if at least one eye was graded to have TF and likewise for the photo grade. In the event that grading data were available for only one eye, and if that eye did not have TF, the overall TF grade at the child level was designated as missing for that child because the status of the fellow eye could either be TF or no TF. Statistical analyses were performed using STATA 15 (StataCorp, College Station, TX).

## Results

### Pilot test at Wilmer eye institute

All images were gradable, the conjunctival vessels were easily seen, and the “loupes-view” magnification window allowed for successful proprioception. However, because there was no active trachoma in our Baltimore-based sample, a field test in children in a trachoma endemic area was necessary.

### Pilot field test in Tanzania

The intergrader agreement in the field between the ophthalmologist and field grader for TF was excellent (kappa = 0.78). A comparison of the of the same individual’s field grade and grade of the images taken using ICAPS was lower (kappa = 0.62 for the ophthalmologist and 0.58 for the field grader). We determined after discussion with the ophthalmologist and the field grader that the lower agreement was due to difficulties both had in identifying follicles in the images. Based on this experiment, further adjustments to the focus and resolution of the image were made to optimize the image quality.

#### District survey field test

A total of 1305 children participated in the study, contributing 2610 eyes for analyses. Of these, 2373 eyes had gradable images and 237 eyes (9.1%) did not. Of the 1305 children, 1106 (84.8%) either had gradable images in both eyes or there was TF in the one eye that had a gradable image. The remaining 199 children (15.2%) thus had either both eyes with ungradable images, or one eye with ungradable images and the other eye graded as “No TF”. Of these 199 children contributing 398 eyes, 237 eyes were ungradable and 161 were graded as “No TF”.

**Fig 3:**
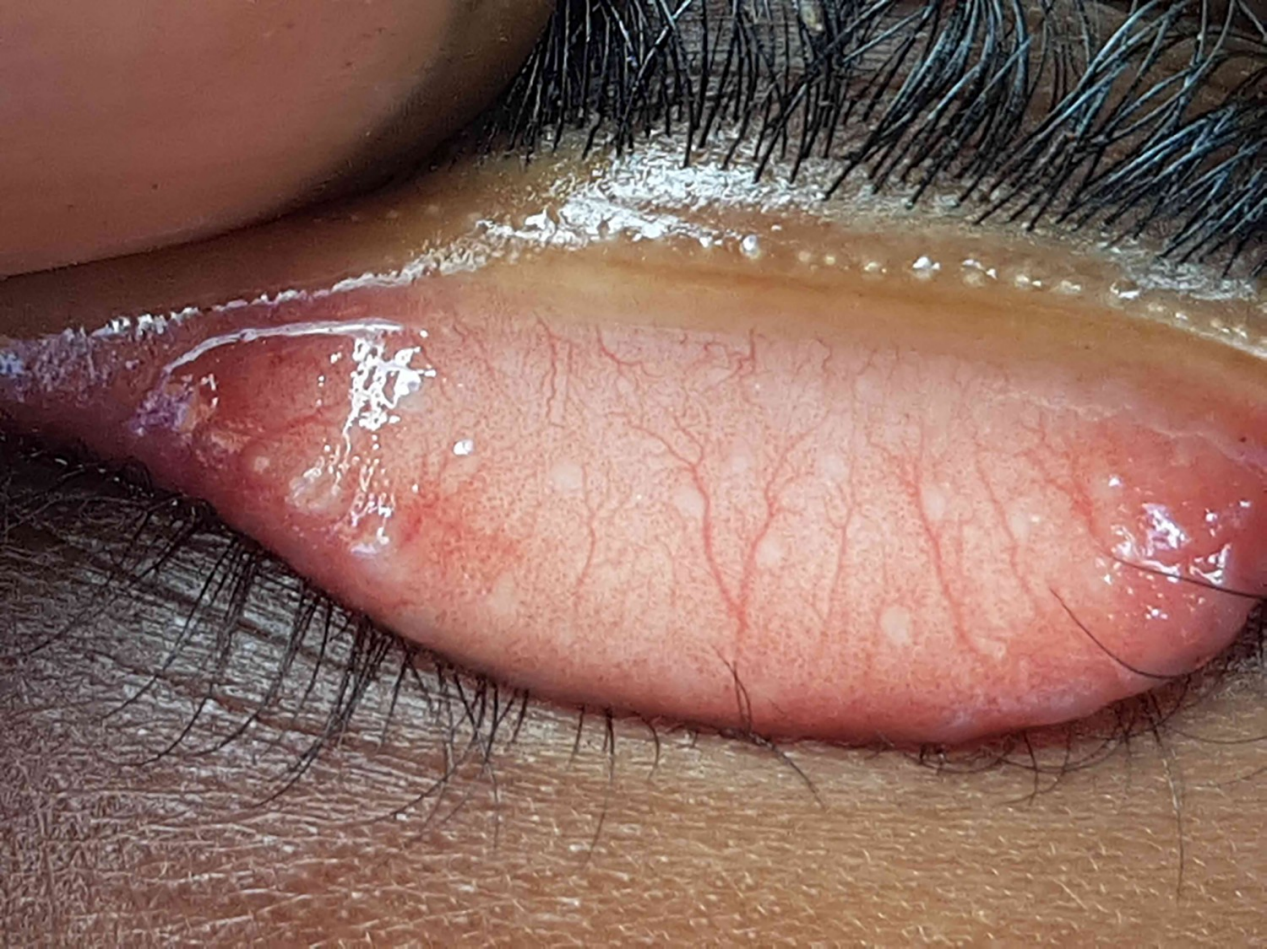
Example of a lid image taken by ICAPS.

**Fig 4:**
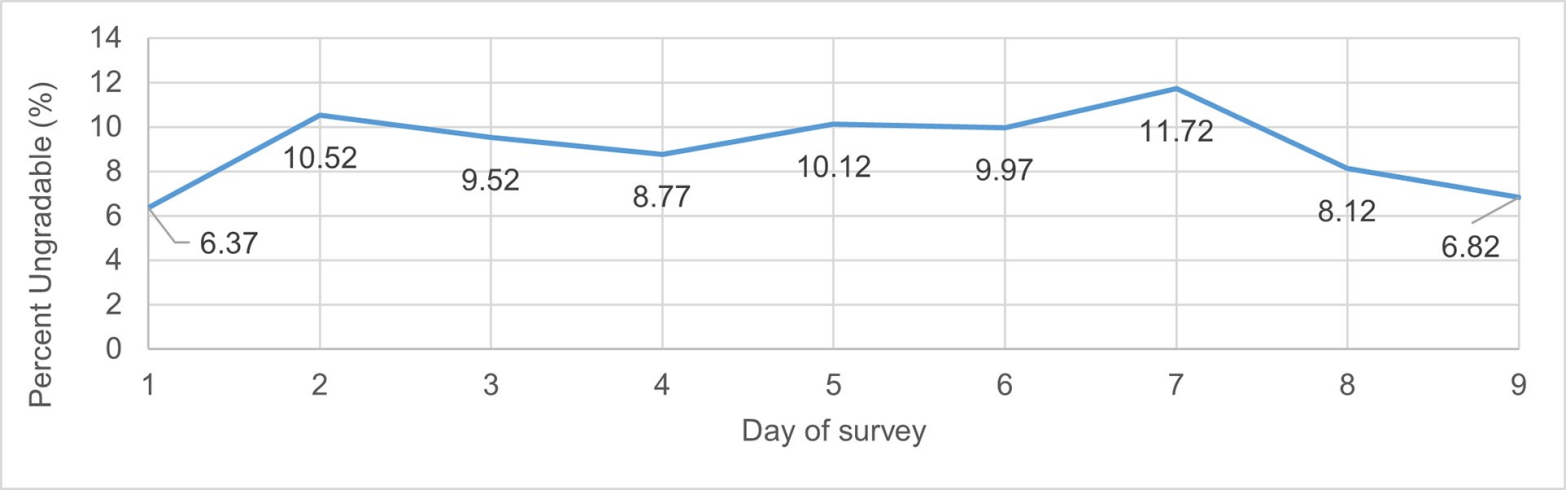
Percentage of ungradable images over the survey duration.

Most images were gradable and of good quality, an example of which is shown in Fig 3. More ungradable images occurred in children ages 1–3 years (18.5%) compared to older children, 4–6 years (5%) and 7–9 years (3.2%) of age (p = <0.001) (Table 1). The rate of ungradable images was essentially constant over the study period suggesting no strong learning effect (Fig 4).

**Table 1:** Percentage of ungradable images of the 2610 eyes by age group.

Age	Ungradable images
**1–3 years**	**18.5% (164/888)**
**4–6 years**	**5.0% (49/980)**
**7–9 years**	**3.2% (24/742)**
**Total**	**9.1% (237/2610)**

χ^2^ (2, N = 2610) = 145.3, p<0.001

Among the gradable images, 4.5% of eyes had TF by field grade, and among the ungradable images, 5.5% had TF by field grade, which was not a statistically significant difference (p = 0.494) (Table 2).

**Table 2:** Field-assessed prevalence of TF in gradable vs ungradable images in the 2610 eyes.

	Gradable images (n = 2373)	Ungradable images (n = 237)
**No TF in field**	2266 (95.49%)	224 (94.51%)
**TF in field**	107 (4.51%)	13 (5.49%)

χ^2^ (1, N = 2610) = 0.47, p = 0.494

Gradable images were available for 1106 children (564 girls and 542 boys); mean age 5.2 years (SD 2.4). A few children had up to two images for an eye. The overall percentage of TF among these 1106 children by field grade was 5.2%, and by photograph grade was 5.0%. The agreement of field and photograph grade was kappa = 0.53 (95% Confidence interval = 0.40–0.66) (Table 3).

**Table 3:** Comparison of ICAPS grade to field grade for 1106 children with gradable images in survey.

	ICAPS grade = No TF	ICAPS grade = TF	Total
**Field grade = No TF**	1025	24	1049
**Field grade = TF**	26	31	57
**Total**	1051	55	1106

Excluding children whose images could not be graded.

The agreement between field and photograph grade was similar for each age group and greatest for the 4–6 years group (kappa = 0.58) (Table 4).

**Table 4:** Agreement between field and image grade for 1106 children with gradable images by age group.

Age group	Agreement	Kappa (95% Confidence Interval)
**1–3 years**	93.2%	0.48 (0.28 to 0.67)
**4–6 years**	95.3%	0.58 (0.42 to 0.74)
**7–9 years**	97.71%	0.49 (0.18 to 0.79)

The ICAPS performed well in the field, with all three systems functioning properly through the completion of the field test. The batteries and phone were re-charged each evening and the backup batteries enabled us to use each system continuously without downtime, provided the headsets were periodically allowed to go to sleep mode by removing them as the grader/photographer walked between houses.

## Discussion

We created the ICAPS system to respond to a challenge to create a hands-free system that would allow a photographer to evert the lid and capture a quality image, label it, and save it for later photo grading. The evaluation of the system revealed reasonable agreement but also the need for more training to lower the rate of ungradable images and more thought into integration of ICAPS data with data collected using other systems.

In general, an ideal camera system for trachoma surveys should be durable, inexpensive to procure and maintain, and enable well-trained photographers to take good quality pictures of the tarsal conjunctiva quickly with appropriate focus, lighting, and magnification. Conventional digital SLR cameras often require attachable macro lenses to be able to take a close-up image of the tarsal conjunctiva and the photographer often needs a second person to hold the child and evert the lid. Though attachable lenses for smartphones have been tested, certain newer smartphones come with a macro lens embedded though the resolution is reportedly low [7]. The ICAPS system builds on previous technologies to create a hands-free, head-mounted camera system enabling a single person to evert an eyelid, take an image without touching the equipment, label and save it. A custom-built application utilizes software to magnify the field of view and capture the entire eyelid using only the native smartphone camera with high resolution.

The development of the ICAPS went through several iterations. An initial attempt to use voice-activated technology to take the image was not successful because the program was limited to spoken English, was particularly poor for accented English, and was easily disturbed by extraneous noise. We switched to a foot pedal approach which proved robust in our trials. We also discovered a slight delay between trigger actuation and image capture, so photographers required training on holding the child steady for an extra second or two for clarity. A second issue was created by the camera’s native autofocus, which tended to focus on high contrast of the child’s eyelashes rather than the tarsal conjunctiva. We initially disabled autofocus to allow for manual focus on the lid but found a better solution by having the software display a zoomed image of the eyelid within the field of view in the head set window and allow autofocus only on this window. This permitted the autofocus feature to focus on the conjunctiva and improved the clarity of the image. A separate challenge during development was acquiring high enough quality images to grade follicles. This ultimately required iterative programming to generate an image that balanced file size and overall quality without introducing unmanageable image artifacts introduced as a result of the hardware’s original purpose (VR headset with augmented reality features).

File sizes averaged 4–11 MB and were too large to be uploaded efficiently directly from the handset with the bandwidth available in Tanzania. In anticipation of this issue, the reading center developed a web-based tool which could be accessed on a laptop, to compress the images prior to uploading to the cloud server. In this way, we could download the images from each ICAPS system each evening to the laptop and upload them to the cloud server. Despite image compression, which reduced file size by approximately 95%, image upload required greater bandwidth than available using networks in the study team’s base lodging, so purchase of a mobile data card was necessary (12 GB prepaid data plan for around US$6.50). If access to the images is not urgent, this connectivity issue could also be rectified by uploading all images once broadband internet access is re-established after survey completion.

The time taken to grade a child in the field, around 3–5 seconds per eye once the lid was turned, was comparable to taking a photograph and grading the images using ICAPS with one grader. If multiple images were taken and/or multiple graders used to grade the image, the time would be greater. A skilled photographer can capture the entire eyelid in focus with little to no glare or artifacts, so one image per eyelid may suffice. For research purposes, we insist on two images per eyelid, to ensure of at least one image. There is a trade-off between having 2 versus 1 image per eye, as it doubles the storage needs, grader time etc. Initially for the survey, we hoped that a single photograph for each eyelid would suffice but with only one day to train new photographers in using the ICAPS, we permitted two images per eye at the start until the photographers felt more comfortable in the images they were accepting as adequate. The key point is that if there is enough training including ample field experience provided to photographers, we believe that a single photograph of each eyelid could be sufficient, but this is a reasonable operational research question. It should be noted that the most significant time allotment during surveys is often getting to the field and going door to door; the examination time is relatively modest in comparison.

It is difficult for one person to both evert the lid and take an image using a smartphone or digital camera, especially in distressed children, so a hands-free system such as the ICAPS obviates the need for the additional staff to flip the lid as the image is taken. In addition, the hands-free aspect decreases the risk of contaminating the camera system. Using a single photographer to evert the eyelid and take the photograph may reduce the risk of disease transmission due to fewer survey team members, a particularly useful feature to consider in light of the COVID-19 pandemic. However, it should be noted that trachoma prevalence surveys also assess the presence of trachomatous trichiasis (TT), and to date, photography does not reliably capture TT, so the photographer/field grader would still need to assess TT.

The ICAPS proved to be robust in the field and was somewhat successfully linked with existing Tropical Data systems. The training was smooth as the ICAPS was fairly intuitive to operate, particularly to those familiar with the use of a smartphone. However, we found that one day of training did not provide enough time to practice taking high-quality photographs in children. Our initial pilot test experience in Tanzania with members of the Kongwa Trachoma Project team guided our decision to conduct training over a single day, but they were experienced photographers used to imaging children. Clearly, while field graders are very used to examining children, taking images of eyelids by inexperienced photographers in field conditions required more practice. Training multiple photographers, similar to how Tropical Data trains multiple graders, some of whom may not pass certification, would be ideal. That way, the best trainees would be selected for the field allowing for the highest quality images to be taken. In fact, we did replace a grader-photographer in the morning session of the training who was not able to operate the system successfully in the classroom. In addition, we would modify the certification criteria to be more rigorous, insisting on high quality images from 5 children in a row, including at least 3 that were ages 1–3 years.

The field teams anecdotally reported to us a favorable impression after using the ICAPS. The field graders serving as ICAPS photographers were quickly at ease with using the system and its functionality. Despite the limited field experience, the photographers had little difficulty everting the eyelids and taking images using the foot-pedal. They reported some difficulty with determining if the image presented for saving was of sufficient quality, at least at the outset. The photographers suggested that two-person teams were sufficient to divide the tasks required for the survey–one ICAPS photographer and another staff to assist in locating individuals for testing, performing randomization, and tracking GPS coordinates in out-of-network regions. Some key recommendations included having a carrying bag with multiple compartments to separate the ICAPS components, cleaning out the headset at the end of the survey day to prevent dust build-up, and installing screen protectors on the smartphone. The trainers reported that it was somewhat onerous after a long day in the field to collect all the phones and charge them, download the images to the laptop and to upload the images to the reading center server each night. They suggested using someone not in the field each day who could be assigned this task, or if not, that a field team member could download the images and charge the phones each night, and upload the images on off days, or lighter days.

There were very few technical complications that occurred in the field all of which were easily resolved and constitute “lessons-learned” that other investigators in this domain may benefit from. We had a single system that overheated and stopped functioning at the end of a long afternoon. This was a one-time event that occurred because the grader/photographer neglected to remove the ICAPS headgear between houses, which sends the unit into sleep mode. The device resumed optimal functionality once the system cooled down. All equipment was very durable and showed no signs of wear and tear following rigorous field testing. A key logistical factor was that access to electricity at the end of each day was necessary as we needed to recharge the batteries on the phone and the external battery packs every evening.

Another challenge was posed by UPC barcodes. Properly printed without smudging, the barcode was easily readable by the ICAPS system which then automatically assigns this as the ID number to the next set of acquired images. However, the photographer must manually indicate right and left eye, and when the next child is being imaged. Occasionally the photographer forgot to indicate a new person was being imaged or forgot to indicate the correct eye. We had initially written the app to allow only 1 photograph per eye, per barcode to avoid duplicate use, but due to feedback from users this feature was disabled. At the outset, the photographers were uncomfortable submitting only one image if they had concerns about image quality.

Initially, the system forbade reuse of barcodes, but we found clear instances where re-use was permitted. This led to some difficulty integrating data between ICAPS and the Tropical Data system. This was compounded by the fact that Tropical Data uses older phone technology with an unreliable barcode reader, so often the match by barcodes was less successful than planned. Early on in our analysis, we found that nearly half of the image grade data needed to be manually linked to the field grade data. For some linkages, we had to rely on the time stamp on the images to group them correctly. Clearly if photography is to be integrated into Tropical Data, a more robust system of integration will be necessary.

Smartphones have been previously evaluated to detect TF and the results were promising. One study comparing images taken with an iPhone 4 to field grading reported low sensitivity (41%) but high specificity (91%) for TF [8]. Another study evaluated an external attachment which allows the smartphone to take magnified images with greater image pixel-density dedicated to the eyelid [9]. It also used an external magnification attachment with supplementary light sources and a iPhone 4S smartphone, and reported 84.1% sensitivity and 97.6% specificity for TF [7]. Our system had high specificity, 98%, but lower sensitivity, 54%, compared to the field grade, though without a true gold standard assessment, we believe sensitivity and specificity could be misleading statistics.

In fact, we believe there is likely to be uncertainty in both image and field assessment of TF with borderline cases in both directions. It is interesting that the estimated percentage of TF cases in the sample was similar for both the ICAPS and the field grade, 5%. While it may be the result of the low proportion of TF in the sample, it may also reflect a similar and unknown propensity to miss cases by both field and image grading.

There are also issues using kappa as the test of agreement when the prevalence of disease is low. For example, had the proportion of children with TF by field grade been 20% instead of 5%, with the same sensitivity and specificity the kappa statistic for agreement would have increased to 0.62. Though the kappa statistic was below the cutoff used to certify field graders (>0.7), the absolute kappas cannot be compared because the kappa statistic is heavily influenced by prevalence and field grader agreement is recommended using a high prevalence environment [10].

We are working to understand the possible sources of mismatch between field and image grades. In the future, it would be interesting to add more image and field graders, as was done in a study in Ethiopia or use a different analytic approach to determine the ground truth of the grade for each child, such as latent class analyses or a binned approach [9,11].

There was a greater proportion of ungradable images in the study than we had hoped. Of 2610 images, 9.1% were ungradable. Of note, field cases of trachoma were not overrepresented among the ungradable images in our study, so presumably the proportion of cases with TF was not affected, but that may not always be the case. More ungradable images occurred in children ages 1–3 years, likely due to resistance to lid eversion in this age group, and loss of focus due to movements and the small size of the tarsal plate. However, the proportion of ungradable images in the other children was still 3–5%; if the survey is trying to detect a prevalence of TF of less than 5% and the proportion of ungradable images is biased by trachoma status, then the estimated prevalence using gradable images would be biased with programmatic implications. The percentage of ungradable images did not change substantially over the course of the survey as might be presumed with learning effects; however, there was some indication of improvement over the last three days. The lower rate of ungradable images on the first few days was likely due to graders being given permission to take 2 images of an eye and saving them until they learned how to judge the quality of what they were saving. This practice was curtailed after two days because the volume of images to be transmitted and linked over the course of the survey would have been too large. Images were deemed ungradable due to a variety of reasons such as: 1) too much glare on the eyelid, likely exacerbated by tear film; 2) the image of the eyelid was too small; 3) there were obstructions of the grading area; 4) and off-focus images due to movement. It is important to note that all these issues could be minimized with more training with increased field experience allowing for a single high-quality image per eyelid to potentially be sufficient.

There were a few limitations in this study. First, the survey start was delayed so the ICAPS trainers had to leave with their equipment prior to completing the entire district Two villages that were a part of the district survey were therefore not assessed using the ICAPS so the data provided here could not be used to estimate the prevalence of TF in the district. Second, the graders in the field did not also grade the images, so we do not have agreement from their field and image grades. This would have been difficult in any case as the field graders were not identified individually in the Tropical Data reports. Third, the findings from this study may not be applicable to other settings with different prevalence of trachoma and different trachoma graders. Further field tests and operational research would help identify any technical or region-specific issues. Finally, the issue of how to include TT as part of the survey if only photographers are deployed must be resolved.

In conclusion, the preliminary field evaluation of the ICAPS proved it to be an acceptable hands-free method for collecting images in children. Moreover, the system enables the upload of images to a reading center, allowing prevalence surveys for TF to be performed without standardized field graders. The ICAPS can be a useful tool to document cases of TF, not only for potentially assessing prevalence but also to form a repository to educate graders in the future and for consultation using telehealth [12]. Saving images additionally allows for the flexibility to grade the same eyelids multiple times and could be useful to audit field grading even looking for additional signs, which is nearly impossible with only field grading. Artificial intelligence and automated algorithms to grade images may be a potential future alternative to training graders but it is clear that such developments may be years away and further improvements are necessary prior to use [13]. Ultimately, a telemedicine paradigm using photography and a remote reading center may be a more cost-effective method to track TF prevalence especially in regions where training and deploying field graders can be a challenge.

## Supporting information

S1 Data Excel spreadsheet with raw data. (XLSX)
